# Comparative efficacy of epoetin alfa vs. darbepoetin in children with chronic kidney disease: a systematic review, meta-analysis and cost-effectiveness analysis

**DOI:** 10.1007/s40620-025-02303-8

**Published:** 2025-05-11

**Authors:** Nicola Bertazza Partigiani, Alessandro D’Uva, Serena Vigezzi, Alessandra Rosalba Brazzale, Enrico Vidal

**Affiliations:** 1 Pediatric Nephrology, Department of Women’s and Children’s Health, University Hospital of Padua, Padua, Italy; 2 University of Padua, Padua, Italy; 3 Department of Statistical Sciences, University of Padua, Padua, Italy; 4 Department of Medicine (DMED), University of Udine, Udine, Italy

**Keywords:** Anemia, Chronic kidney disease, Children, Erytropoiesis-stimulating agents

## Abstract

**Background:**

Recombinant human erythropoietin (rHuEPO) and darbepoetin alfa (DA) are key treatments for anemia in individuals with chronic kidney disease (CKD), including children, but evidence comparing their efficacy in the pediatric population remains inconclusive.

**Methods:**

This systematic review, adhering to PRISMA guidelines, analyzed randomized controlled trials and observational studies comparing rHuEPO and DA in pediatric patients with CKD (≤ 18 years; ≥ 10 children per study), searched across medical databases and clinical trial registries until 31/12/2024. The Cochrane Risk of Bias was used for assessment. Meta-analysis evaluated hemoglobin (Hb) increase and cost-effectiveness using the incremental cost-effectiveness ratio.

**Results:**

From 1298 screened articles, 7 studies were included: 3 prospective studies, 2 randomized open-label non-inferiority trials, and 2 retrospective cohort studies, comprising 208 children for direct comparisons and 357 for transitioning studies. Meta-analysis found no significant Hb improvement differences between rHuEPO and DA after 21–28 weeks of treatment (DA + 0.15 g/dL, 95% CI − 0.22 to + 0.52). rHuEPO was more cost-effective than DA. Transitioning to DA increased Hb by + 0.93 g/dL (95% CI 0.53–1.33) in children with suboptimal levels, after 21–28 weeks of rHuEPO. The incremental cost-effectiveness ratio of switching to DA was ~ €340 per g/dL of Hb over 24 weeks.

**Conclusions:**

rHuEPO is the most cost-effective initial anemia treatment in pediatric CKD. However, transitioning to DA may be considered for patients who do not achieve adequate Hb response. The small number of randomized controlled trials (RCTs), variability in dose conversion, and study heterogeneity may limit generalizability.

**PROSPERO ID:**

CRD42023460872.

**Graphical abstract:**

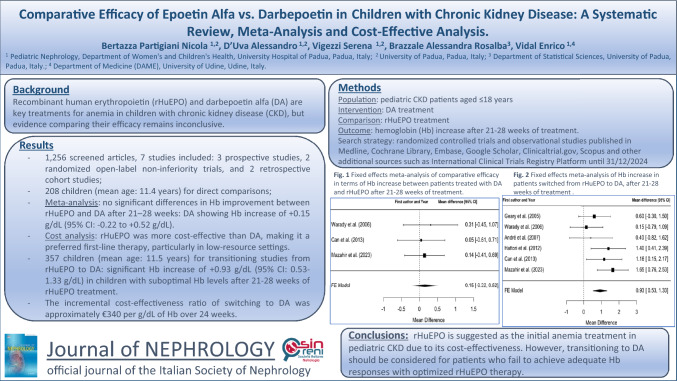

## Background

Chronic kidney disease (CKD) poses a significant medical challenge in children, marked by the progressive decline in kidney function and its associated complications. Anemia, a common and debilitating consequence of CKD in pediatric patients, is primarily caused by impaired erythropoiesis and reduced erythropoietin production due to kidney dysfunction [1] Anemia in children is defined based on the 2012 KDIGO Clinical Practice Guideline for Anemia in Chronic Kidney Disease, with hemoglobin (Hb) cut-off levels varying based on age and sex [2]. Evidence from the North American Pediatric Renal Trials and Collaborative Studies (NAPRTCS) cohort consistently reveals an escalating risk of anemia with the progression of CKD stages. The prevalence stands at 73% in stage 3, 87% in stage 4, and it is over 93% in stage 5 CKD. Moreover, among children receiving erythropoiesis-stimulating agents (ESAs), more than 20% in stage 4 and over 40% in stage 5 CKD exhibit persistently low Hb levels [1, 3].

In children affected by CKD, a low Hb level serves as a robust and standalone predictor of mortality and is linked to a higher frequency of hospitalization, both in the dialyzed and predialysis population. Anemia emerges as a significant cardiovascular risk factor in children, increasing the likelihood of developing left ventricular hypertrophy (LVH) independently of blood pressure, even in cases of mild to moderate CKD [4]. Anemia has a profound impact on the quality of life in these patients, highlighting the critical importance of timely and appropriate treatment to improve a wide range of outcomes [4].

ESAs have revolutionized the management of anemia in pediatric CKD. Their efficacy in increasing Hb levels and improving overall quality of life has been well-documented in adult CKD patients [5, 6].

The Anemia Workgroup of the National Kidney Foundation's Kidney Disease Outcomes Quality Initiative (KDOQI) recommends a target hematocrit range of 33–36% (corresponding to an Hb level of 11–12 g/dl) for both children and adults. Achieving this target often necessitates the use of recombinant human erythropoietin (rHuEPO) [7–9]. RHuEPO and darbepoetin alfa (DA), a long-acting ESA, are critical in directly stimulating erythropoiesis in patients with CKD. These therapies significantly reduce the reliance on blood transfusions, thereby minimizing associated risks such as iron overload, alloimmunization, and transfusion-related infections [10–12]. Their role is particularly vital in pediatric patients with kidney failure, for whom long-term management of anemia is crucial to improve overall quality of life and clinical outcomes. Additionally, the use of these agents aligns with strategies to preserve access to future kidney transplantation by reducing sensitization to human leukocyte antigens [13].

While ESAs provide significant benefits in managing anemia in children with CKD, concerns persist regarding their long-term safety and the establishment of optimal dosing strategies [14, 15]. Current evidence does not clearly define whether rHuEPO or DA should be the first-line treatment, leaving the choice of therapy largely dependent on factors such as patient-specific needs, pharmacokinetics, physician preference and dosing convenience [9, 16].

This review aims to provide a comprehensive analysis of the use of rHuEPO and DA in children with CKD. The primary goal is to assess whether either medication demonstrates superior efficacy in improving and maintaining Hb levels in this population. Additionally, the review seeks to perform a cost-effectiveness analysis to identify the most beneficial treatment strategy, with careful consideration of economic factors.

## Methods

Our study conforms to the Preferred Reporting Items for Systematic Reviews and Meta-analysis guidelines [17].

The protocol for the study was registered on PROSPERO on October 3, 2023 (ID: CRD42023460872) and was updated on January 7, 2025, to reflect the status of the systematic review. No major changes were made to the protocol.

### Inclusion and exclusion criteria

Randomized controlled trials (RCTs) and observational studies, both prospective and retrospective, comparing rHuEPO and DA in children (aged 18 years or younger) diagnosed with CKD and anemia were included in this systematic review. The studies had to indicate dose and administration regimen to be included.

CKD was defined according to the KDIGO 2021 Guidelines as abnormalities of kidney structure or function (estimated glomerular filtration rate (eGFR) < 90 ml/min/1.73 m^2^), present for > 3 months, with implications for health. Patients on kidney replacement therapy were also included [18].

We excluded studies based on design (case report, review, animal models or editorials), or patient populations (patient > 18 years) and number of patients (< 10 children).

### Outcome measures

In our study, we evaluated the efficacy of both rHuEPO and DA by examining their impact on both primary and secondary endpoints. The primary outcome measures focused on achieving a minimum increase of 1 g/dl in Hb levels from baseline or the ability to maintain Hb levels within the range of 11–12 g/dl [2]. Secondary outcome measures included needing blood transfusion, switching protocols from rHuEPO to DA, and cost-effectiveness analysis.

### Search strategy

A comprehensive literature search was conducted to identify relevant studies. Databases such as Medline (https://pubmed.ncbi.nlm.nih.gov), Cochrane Library (https://www.cochranelibrary.com), Embase (https://www.embase.com), Google Scholar (https://scholar.google.com), Clinicaltrial.gov (https://clinicaltrials.gov), Scopus (https://www.scopus.com/home.uri) and other additional sources such as International Clinical Trials Registry Platform (https://www.who.int/clinical-trials-registry-platform), were systematically searched until 31/12/2024 according to the Cochrane Handbook for systematic review. The search terms included epoetin alfa, darbepoetin, CKD, anemia, and children; a combination of boolean operators was used to refine the search. The research methodology is provided in the supplementary materials.

### Screening process

Two independent reviewers (S.V. and N.B.P.) conducted the initial screening of titles and abstracts to identify potentially eligible studies. Full-text articles were then assessed for eligibility based on the predefined inclusion criteria by the same two investigators (S.V. and N.B.P.) who were not blinded to the journal name, institution or study authors. Discrepancies were resolved through consultation with a third reviewer (A.D.).

### Data extraction

Data were extracted from included studies using a standardized data extraction form in Excel sheets. Two tables were used, one for studies comparing patients treated with rHuEPO and DA and one for studies in which the centers switched from using rHuEPO to DA. Type of study, eligibility criteria, number of patients, loss to follow up, endpoint, number of patients on dialysis, duration of therapy (weeks), number of rHuEPO-naive patients, Hb increase, drug dosage and frequency of administration and need for blood transfusion were extracted.

### Quality assessment and risk of bias

We assigned a quality measure to each of the included studies, using the National Institutes of Health Quality Assessment Tool for Controlled Intervention Studies or for Observational Cohort and Cross-Sectional Studies 14-item checklists [17, 19].

Based on this rating system, two investigators (S.V. and N.B.P.) rated each study overall as either poor, fair, or good. Then, we calculated the percentage of overall agreement between the two independent reviewers’ assessments of study quality. Disagreements between investigators about study quality were solved through consensus with a third investigator (A.D.). The risk of bias in RCTs was assessed using the Cochrane Risk of Bias tool. Studies were rated as low, unclear, or high risk of bias in various domains (e.g., randomization, blinding, attrition). The same investigators (S.V. and N.B.P.) independently rated the studies with no disagreements. Publication bias was assessed by N.B.P. using funnel plots to evaluate potential asymmetry in the distribution of studies. Quality assessment and risk of bias are included in the supplementary materials.

### Statistical analysis

Articles which presented a mean difference in terms of Hb after treatment with rHuEPO and DA or after switching from rHuEPO to DA were included in a meta-analysis. A homogeneity test based on the *Q* statistic was performed to evaluate the between-study heterogeneity, which is summarized using the *I*^2^ index. Where significant at the 0.01 level, the summary effect, with a corresponding 95% confidence interval (CI), was obtained from a random-effects model. Publication bias was assessed by an asymmetry of funnel plots. All analyses were carried out using the numerical computing environment R version 4.1.2 (2021-11-01) [20].

Cost-effectiveness analysis was conducted using the incremental cost-effectiveness ratio, calculated as the difference in cost divided by the difference in effectiveness between DA and rHuEPO in terms of their impact on Hb levels [21]. The analysis was conducted based on the cost of equivalent doses of the two drugs (200 IU of rHuEPO and 1 mcg of DA) and the cost of subcutaneous administration, according to our intrahospital price list of services (Table 1). We considered a 24-week period, which is the median duration of the included studies, and analyzed two different administration schedules:

- Once a week for DA and three times a week for rHuEPO;
- Once every two weeks for DA and once a week for rHuEPO.

**Table 1:** Unit cost of drugs and injection used for cost-effective analysis based on the intrahospital price list of services

	Unitary cost (Euros)
rHuEPO (Binocrit 4000 UI)	3.60
DA (Aranesp 20 mcg)	29.70
Subcutaneous injection	3.00

*rHuEPO* epoetin alfa, *DA* darbepoetin

The secondary outcome of blood transfusions was reported as the proportion of patients requiring at least one transfusion during the study period, comparing the incidence between the rHuEPO and DA treatment groups.

## Results

**Fig. 1:**
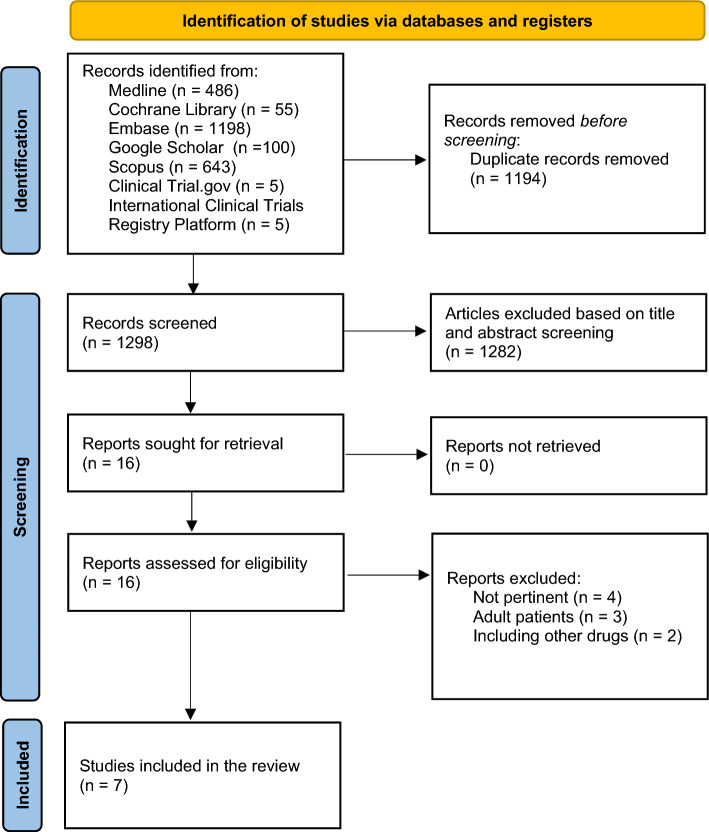
PRISMA flow diagram of study selection

The search retrieved a total of 486 references from Medline, 55 from Cochrane Library, 1198 from Embase, 100 from Google Scholar, 643 from Scopus, 5 from Clinical Trial.gov, 5 from the International Clinical Trials Registry Platform. Following the removal of duplicates, a total of 1298 articles underwent screening based on their titles and abstracts. We identified 16 studies for full-text review and 7 studies were included. Studies are presented according to the outcome considered (Fig. 1).

### Comparative efficacy of DA and rHuEPO

**Fig. 2:**
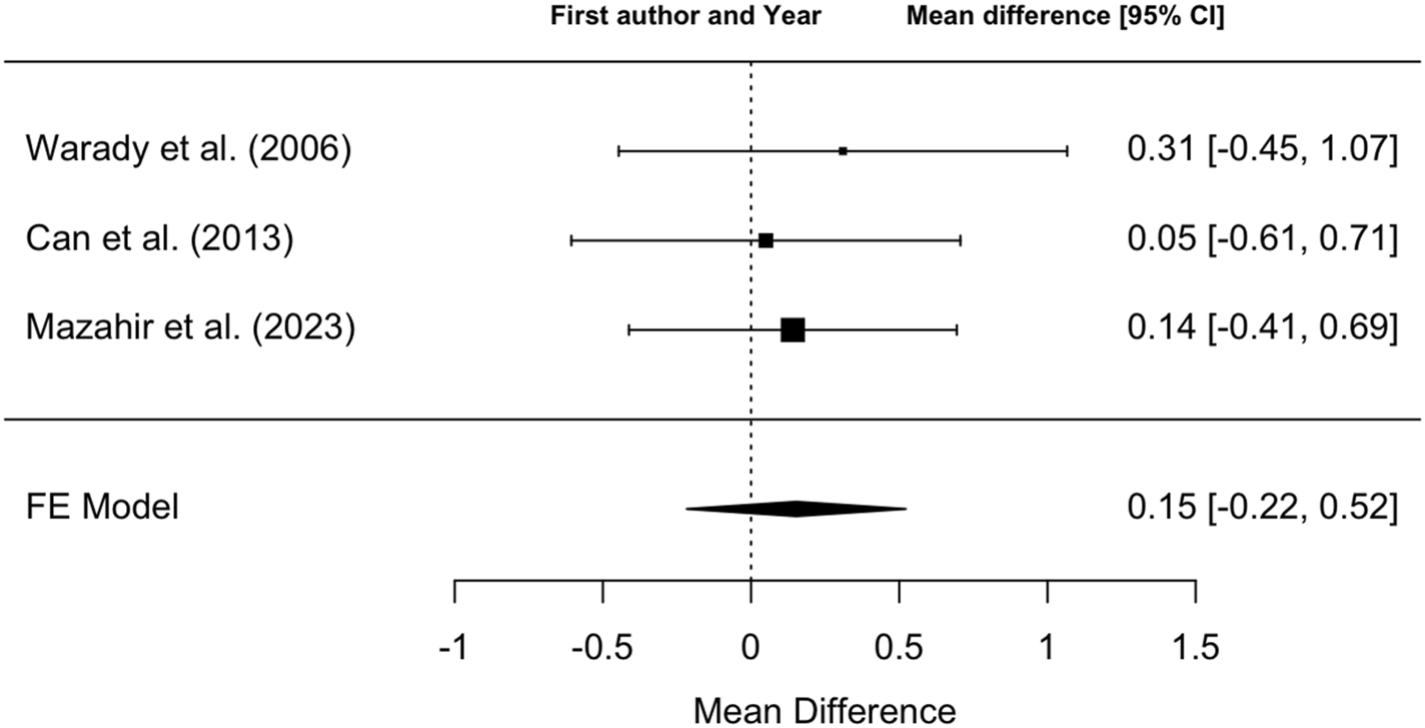
Fixed effects meta-analysis of comparative efficacy in terms of Hb increase between patients treated with DA and rHuEPO after 21–28 weeks of treatment. The funnel plot demonstrated good homogeneity among the studies (*I*^2^ = 0%, *p* = 0.88) (Fig. 3)

Three studies evaluating the comparative efficacy of DA and rHuEPO were included in the systematic review, and their characteristics are detailed in Table 2. Two studies were randomized, open-label, non inferiority trials [22, 23], while the remaining study was a retrospective parallel cohort analysis [24]. Two studies were single center [23, 24] and one is multi-center [22]. A total of 208 children, with a mean age of 11.4 years (± 4.7 standard deviation [SD]), affected by CKD-related anemia were included,. Among them, 137 children (65.9%) were undergoing kidney replacement therapy (65.9%). In the study by Warady et al. [22], 29 patients were lost to follow-up. The primary reasons for discontinuation included kidney transplants (17% for rHuEpo and 11% for DA) and adverse events (0% for rHuEpo and 5% for DA). The other two studies [23, 24] did not report any withdrawals during follow-up. Two studies [22, 23] included pediatric patients under the age of 18 years with CKD ranging from stage 3 to kidney failure, who received rHuEPO for 8 weeks prior to randomization. In contrast, Can et al. [24] included pediatric patients with CKD-related anemia, regardless of CKD stage, who were treated with either rHuEPO or DA for a minimum of 6 months. Overall, 125 patients were treated with DA, of whom 106 completed the study, spanning a period of 21–28 weeks (Table 3). In comparison, the rHuEPO group included 83 patients, with 73 completing the study period. Regarding the mean increase in Hb levels, patients in the DA group achieved an average increase of 0.93 g/dl (SD ± 0.23), whereas those in the rHuEPO group experienced an average increase of 0.76 g/dl (SD ± 0.24 g/dl). All three studies consistently indicated no significant difference in efficacy between DA and rHuEPO for the treatment of CKD-related anemia in children. Our meta-analysis further supports this finding, showing no significant difference between the two treatments. Specifically, DA was not associated with a significant Hb increase of + 0.15 g/dl (95% confidence interval [CI] − 0.22 to + 0.52 g/dl) after 21–28 weeks of treatment (Fig. 2).

**Table 2:** Study types, including criteria and populations analyzed for comparative efficacy between rHuEPO and DA

Author	Year	Journal	Nation	Type of study	Number of centers involved	Time period considered	Population			
							Inclusion criteria	Number of patients	Mean age (years)	Patients lost to follow up
Warady et al.	2006	Pediatric Nephrology	USA	Randomized, open-label, non inferiority trial	Multi-center	NA	Age 1–18 years, eGFR < 30 ml/min/1.73 m^2^, receiving rHuEpo for 8 weeks prior to randomization	124	12 ± 5	29
Mazahir et al.	2023	European Journal of Pediatrics	India	Randomized, open-label, non inferiority trial	Single Center	February 2018—January 2019	Age 1–18 years, CKD stage 3–5 and eGFR < 60 ml/min/1.73 m2, receiving rHuEpo for 8 weeks prior to randomization, Hb 9–12 g/dL, TSAT > 20%, ferritin > 100 ng/ml	50	10.7 ± 4.9	0
Can et al.	2013	Pediatrics International	Turkey	Retrospective, double-cohort	Single Center	April 2008—April 2009	rHuEpo or DA for at least 6 months in patients affected by CKD secondary anemia	34	11.42 ± 4.1	NA

rHuEPO: epoetin alfa

DA: darbepoetin

CKD: chronic kidney disease

NA: not available

*eGFR* estimated glomerular filtration rate, *TSAT* transferrin saturation

**Table 3:** Results of included studies analyzing comparative efficacy between rHuEPO and DA

Author	*N* on dialysis	Duration therapy (weeks)	N DA group	*N* completed studies in DA group	Hb increase in DA group (g/dl)	Standard error in DA group (g/dl)	*N* rHuEpo group	*N* completed studies in rHuEpo group	Hb increase in rHuEpo group (g/dl)	Standard error in rHuEpo group (g/dl)	Mean difference	Total standard error	CI		*N* blood transfusions in DA group	N blood transfusions in rHuEpo group
													Min	Max		
Warady et al.	108	21–28	82	63	0.15	0.23	42	32	− 0.16	0.31	0.31	0.38	− 0.45	0.38	5	6
Mazahir et al.	20	24–28	26	26	1.48	0.20	24	24	1.34	0.20	0.14	0.28	− 0.439	0.719	2	2
Can et al.	27	24	17	17	1.16	0.26	17	17	1.11	0.21	0.05	0.33	NA	NA	0	0

*rHuEPO* epoetin alfa, *DA* darbepoetin, *N* number of patients, *CI* confidence interval, *NA* not available

**Fig. 3:**
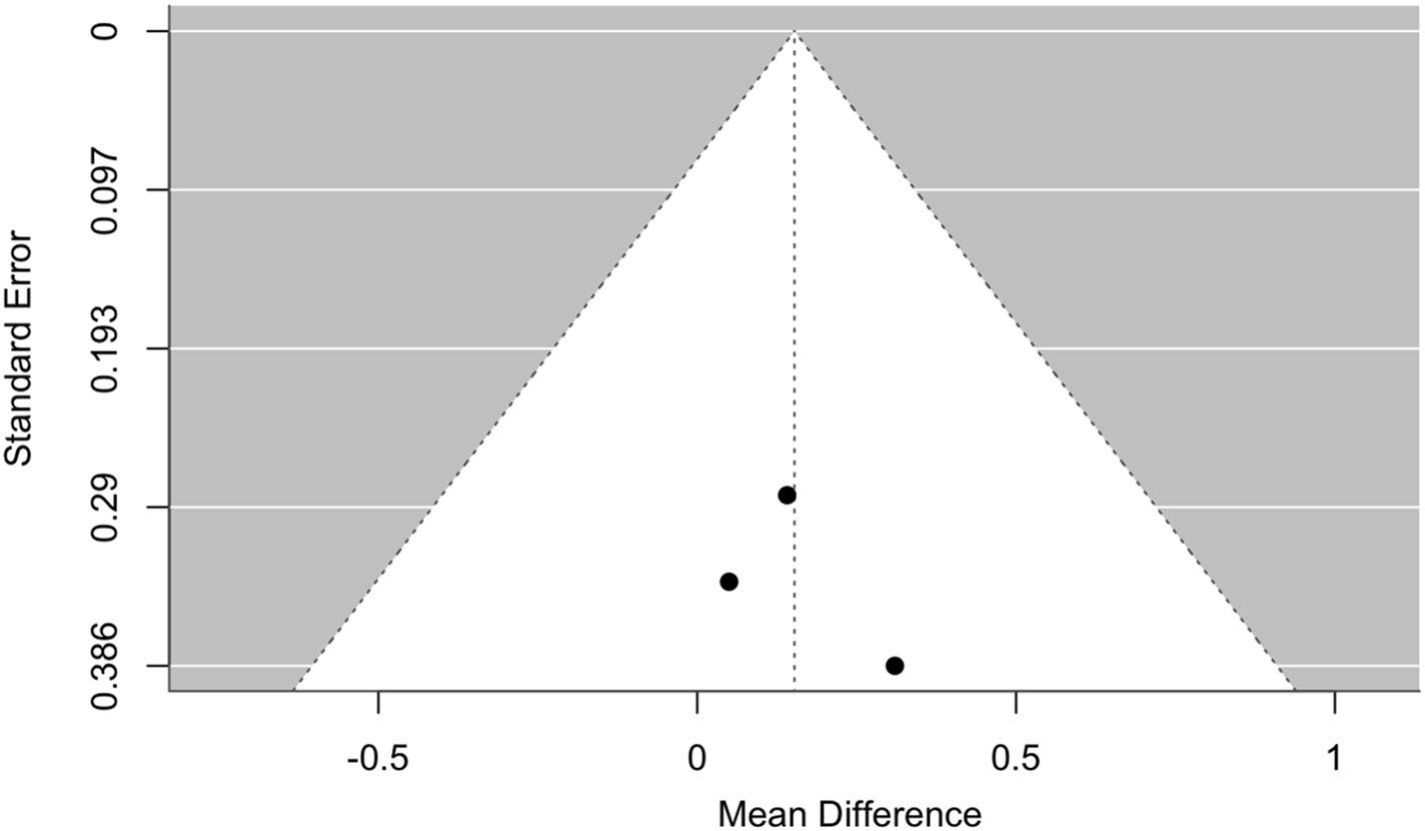
Funnel plot of meta-analysis of comparative efficacy in terms of Hb increase between patients treated with DA and rHuEPO after 21–28 weeks of treatment

Due to the heterogeneity in Hb target definitions across studies, a meta-analysis of the percentage of patients achieving the target Hb range was not feasible. In the study by Warady et al. [22], the mean percentage of Hb values within the target range (10.0–12.5 g/dl) was 73% for rHuEPO and 75% for darbepoetin alfa. Mazahir et al. [23] reported that 62.5% of rHuEPO patients and 69.2% of DA patients maintained Hb levels within the 11–13 g/dl range, with a comparable proportion of patients having Hb levels above 10 g/dl (79% vs. 84%). In the study by Can et al. [24], 6 months after initiation, 29.4% of rHuEPO patients and 17.6% of DA patients achieved Hb levels within the 11–12 g/dl range (*p* = 0.14).

Additionally, 5.9% of patients treated with DA required red blood cell transfusions, compared to 10.6% of those treated with rHuEPo. However, this difference was not statistically significant (*p* = 0.20) (Table 3).

### Switch from rHuEPO to DA

Three prospective studies [25–27], two randomized, open-label, non-inferiority trials [22, 23], one retrospective parallel cohort study [24] and one retrospective cohort study [28] were included in the analysis, each investigating the feasibility, tolerance and efficacy of transitioning from rHuEPO to DA. Four studies are multi-center [22, 25–27], whereas three were conducted at a single center [23, 24, 28]. The general study data and primary outcomes are reported in Table 4.

**Table 4:** Type of study, inclusion criteria and endpoints of included studies which analyzed switching from rHuEPO to DA

Author	Year	Journal	Nation	Type of study	Number of centers	Time period considered	Inclusion criteria	Population	Endpoint
Hattori et al.	2012	Clinical and Experimental Nephrology	Japan	Prospective, single-arm study	Multicenter	January 2009–December 2011	Patients receiving PD aged between 1 and 18 years	28	The primary endpoints were the Hb profiles, which comprised changes in Hb concentration, the rate of increase in Hb concentration, the percentage and time needed to reach the target Hb concentration, and the percentage of patients who maintained the target Hb concentration. Changes in Hb and changes in DA dose per week were also analyzed in some patients who underwent a change in the initial dosing frequency to once every 4 weeks
Hattori et al.	2013	Clinical and Experimental Nephrology	Japan	Open-label, prospective study	Multicenter	October 2010–March 2012	Age 2–18 years, baseline Hb concentration of < 11.0 g/dl for ESA-naıve patients and 9 = < Hb < 12 g/dl for patients switched from rHuEPO (previously treated with rHuEPO)	34	The efficacy of DA was evaluated by the Hb profiles of the patients, i.e. changes in Hb concentration, changes in DA doses per week, rate of increase in Hb concentration in treatment-naıve patients, changes in Hb concentration in patients switched from rHuEPO, and the percentage of patients who maintained the target Hb. Additionally, changes in Hb and changes in DA dose per week were analyzed in some patients whose dosing frequency was switched from once every 2 weeks to once every 4 weeks
André et al.	2007	Pediatric Nephrology	France	Observational, prospective study	Multicenter	January 2003–April 2004	11 years and 18 years with kidney failure, in stable condition, in dialysis or not, pretreated or not with r-HuEPO, TSAT ≥ 20%. and/or serum ferritin ≥ 50 μg/l [	54	Hb at M6
Geary et al.	2005	Kidney international	Canada	Prospective and retrospective study	Single center	NA	< 18 years; eGFR < 30 or kidney failure requiring dialysis, weight > 8 kg	33	The proportion of subjects with mean Hb level > 10.0 g/dL based on all values measured for each patient between 8 and 12 and 20 and 28 weeks; (2) the percentage of all Hb values that were > 10.0 g/dL
Warady et al.	2006	Pediatric Nephrology	USA	Randomized, open-label, non inferiority trial	Multicenter	Not reported	Age 1–18 years, eGFR < 30 ml/min/1.73 m^2^, receiving rHuEpo for 8 weeks prior to randomization	124	Mean change in Hb concentration between the baseline and the evaluation period (weeks 21–28); Secondary endpoints included the percentage of Hb values within the target range (10.0–12.5 g/dl) for each subject and the dose of study drug administered during the evaluation period
Mazahir et al.	2023	European Journal of Pediatrics	India	Randomized, open-label, non inferiority trial	Single center	February 2018–January 2019	Age 1–18 years, CKD stage 3–5 and eGFR < 60 ml/min/1.73 m^2^, receiving rHuEpo for 8 weeks prior to randomization, Hb 9–12 g/dL, TSAT > 20%, ferritin > 100 ng/ml	50	Mean change in Hb between baseline and the evaluation period (24–28 weeks). Secondary outcomes included the incidence of treatment-related adverse events in both groups during the study period and the dose of study drug during the evaluation period
Can et al.	2013	Pediatrics International	Turkey	Retrospective, double-cohort	Single center	April 2008–April 2009	rHuEpo or DA for at least 6 months in patients affected by CKD secondary anemia	34	To compare the clinical efficacy and safety of rHuEPO and DA in the treatment of anemia in children with CKD. target Hb was 11–12 g/dL, ferritin was > 100 ng/mL and transferrin saturation was > 20%. serum ferritin level > 100 ng/mL

*rHuEPO*: epoetin alfa, *DA*: darbepoetin, *Hb*: hemoglobin, *CKD*: Chronic kidney disease, *eGFR*: estimated glomerular filtration rate, *NA*: not available, *PD*: peritoneal dialysis, *TSAT*: transferrin saturation,

A total of 357 patients were included across the studies, with a mean age of 11.5 years (SD ± 4.4 years). Of these, 9 were deemed ineligible for participation, and 67 were lost to follow-up, primarily due to kidney transplantation and, to a lesser extent, adverse events or withdrawal of consent. The studies included a total of 239 patients undergoing dialysis (66.9%), with 128 (53.6%) on hemodialysis (HD) and 111 (46.4%) on peritoneal dialysis (PD).

The 7 studies demonstrated heterogeneous inclusion criteria. Three studies included rHuEPO-naive participants [26–28], and individuals across all stages of CKD were represented. Additionally, three studies enrolled both dialysis and non-dialysis patients [26–28], with varying Hb thresholds documented for study participation (Table 4). Specific levels of transferrin saturation (TSAT) [23, 25, 27], serum ferritin [23, 25, 27], and body weight [28] were required as inclusion criteria in 3 studies. Furthermore, 2 studies [22, 23] included only patients who had been treated with rHuEPO for at least 8 weeks prior to switching to DA.

The primary focus of the studies was to evaluate patients’ Hb profiles over a 24- to 28-week period, assessing parameters such as changes in Hb concentration over time, and the rate of Hb increase. Additionally, the studies examined the percentage of patients maintaining target Hb levels throughout the treatment period and the time required to reach these targets. However, the studies used different Hb target ranges, making direct comparisons challenging. The efficacy of DA was specifically assessed by analyzing changes in Hb concentration, variations in weekly DA doses, and differences in Hb response between treatment-naive patients and those transitioning from rHuEPO therapy. The primary outcomes of each study are summarized in Table 4.

Across all studies, a total of 288 patients completed the study protocol. Among them, 27 patients were rHuEPO-naive and were included in three studies [26–28], while 219 patients underwent a switch in therapy from rHuEPO to DA (Table 5). Two studies [25, 27] reported the pre-switch rHuEPO dose prior to DA, with a mean dose of 126 ± 93 IU/kg/week. For the purpose of our analysis, rHuEPO-naive patients were excluded, as they were not relevant to the primary research objective. In three studies [25–27], the transition from rHuEPO to DA was managed by calculating the DA dose as 1 μg for every 200 IU of previously administered rHuEPO. In one study [28], the initial DA dose was set at 0.45 μg/kg/week, while in two other studies [22, 23], the initial DA dose was 0.42 μg/kg/week. One study [24] did not report the conversion factor between units of rHuEPO and DA.

**Table 5:** Results of included studies which analyzed switch from rHuEPO to DA

Author	Year	Population				Number of patients on dialysis (HD/PD)	Duration therapy (weeks)	*N* of rHuEPO naive	*N* switched to DA	Hb baseline in switched group (g/dl)	Hb end of the study switched group (g/dl)	Switch dose considered
		*N*	Mean age	Lost to follow up	*N* completed studies							
Hattori et al.	2012	28	11.2 ± 5.7	5	23	28 (HD 0/PD 28)	28	0	28	9.9 ± 1.0	11.3 ± 0.9	1 ug DA for 200 IU rHuEPO
Hattori et al.	2013	34	10.4 ± 4.7	10	24	15 (HD 2/PD 13)	24	9	15	NA	NA	1 ug DA for 200 IU rHuEPO
André et al.	2007	54	15.2 ± 2.1	15	39	23 (HD 17/PD 6)	24	10	29	11.1 ± 1.2	11.5 ± 1.7	1 μg DA for 200 IU rHuEPO
Geary et al.	2005	33	9.7 ± 4.4	10	23	18 (HD 7/PD 11)	28	8	22	10.5 ± 1.0	11.1 ± 0.7	0.45 ug DA for 100 IU rHuEPO
Warady et al	2006	124	12 ± 5	29	95	108 (HD 80/PD 28)	28	0	82	11.4 ± 0.6	0.15 ± 0.23	0.42 μg DA for 100 IU rHuEPO
Mazahir et al.	2023	50	10.7 ± 4.9	7	50	20 (HD 13/PD 7)	28	0	26	9.517 ± 0.382	11.165 ± 0.966	0.42 ug DA for 100 ug rHuEPO
Can et al.	2013	34	11.42 ± 4.05	NA	34	27 (HD 9/PD 18)	24	0	17	9.19 ± 0.68	10.35 ± 0.85	NA

*rHuEPO* epoetin alfa, *DA* darbepoetin, *N* number of patients, *NA* not available, *HD* hemodialysis, *PD* peritoneal dialysis

**Fig. 4:**
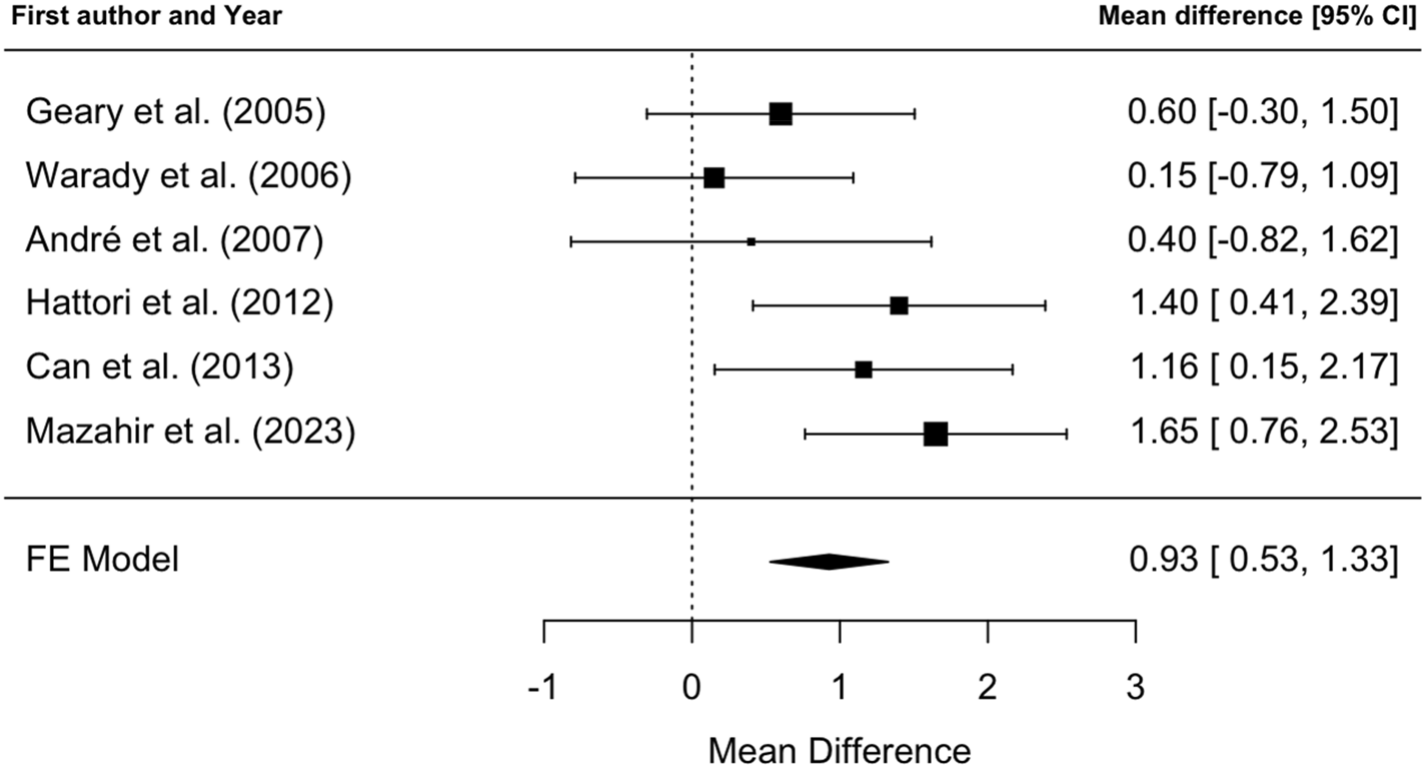
Fixed effects meta-analysis of Hb increase in patients switched from rHuEPO to DA, after 21–28 weeks of treatment. The funnel plot indicated acceptable homogeneity among the studies (*I*^2^ = 32%, *p* = 0.19) (Fig. 5)

Our meta-analysis demonstrated a significant Hb increase of + 0.93 mg/dl (95% CI 0.53–1.33 mg/dL) in patients switched from rHuEPO to DA after 21–28 weeks of treatment (Fig. 4).

**Fig. 5:**
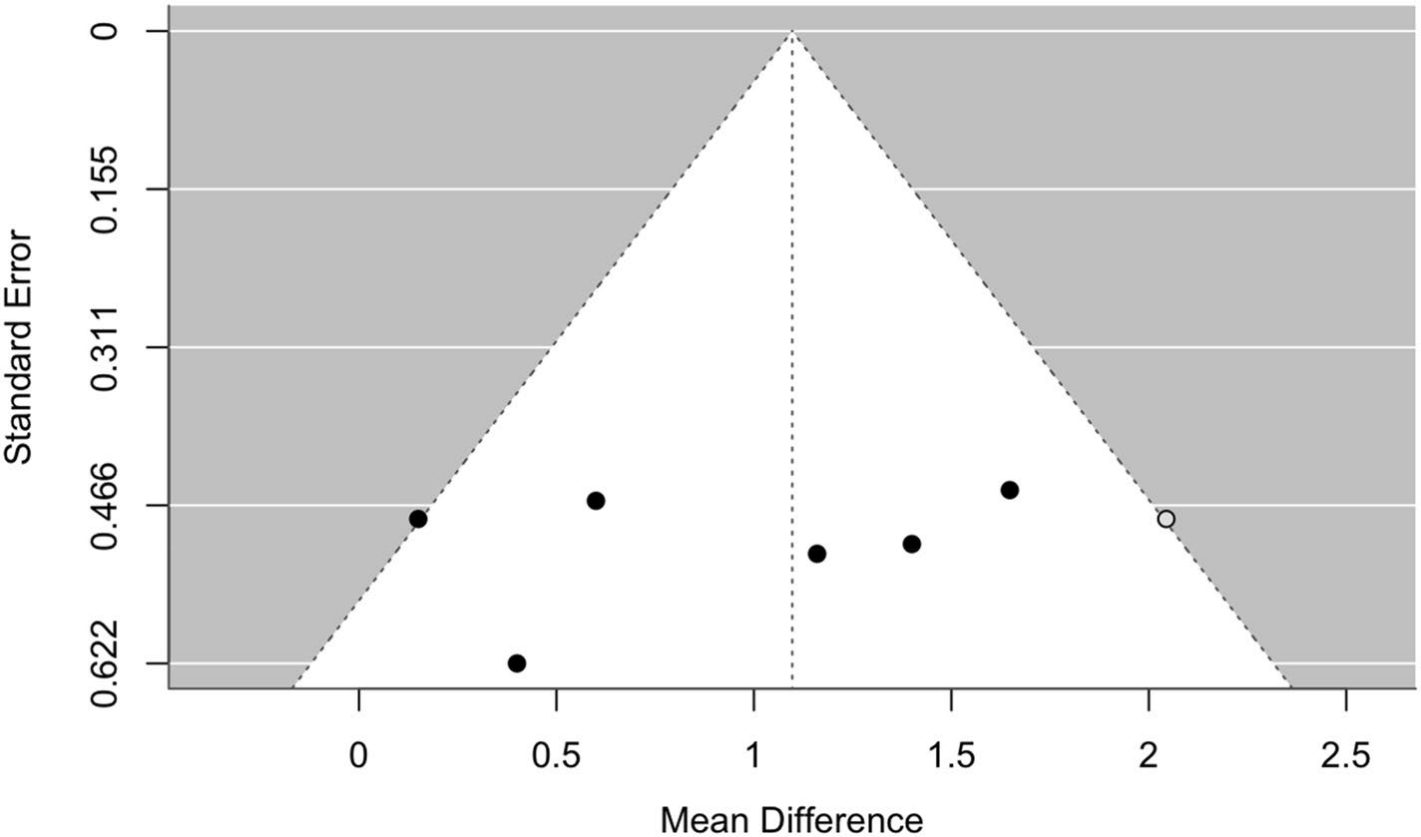
Funnel plot of meta-analysis of Hb increase in patients switched from rHuEPO to DA, after 21–28 weeks of treatment

One study [26] failed to distinguish the Hb increment between the rHuEPO-naive group and those who switched. Consequently, these data were excluded from the analysis. Overall, 65.1% of patients maintained an Hb target within the range of 11–13 g/dl, a percentage that increased to 83% when considering the broader range of 10–13 g/dl. However, Can et al. did not report the percentage of patients achieving Hb levels within the target range [24].

### Cost-effectiveness analysis

The cost of therapy over a 24-week period, considering a single unit of medication, varies depending on the administration schedule:

- Weekly administration: one administration per week of 20 mcg of DA (784.8 euros) compared to three administrations per week of 4000 IU of rHuEPO (470.4 euros), resulting in a cost difference of 314.4 euros.
- Biweekly administration: one administration every two weeks of 20 mcg of DA (392.4 euros) compared to one administration per week of 4000 IU of rHuEPO (158.4 euros), resulting in a cost difference of 234 euros.

The difference in terms of efficacy between these treatments is 0.15 mg/dl (95% CI − 0.22 to + 0.52) of Hb over 24 weeks. The cost-effectiveness analysis for these administration schedules is as follows:

- Incremental cost-effectiveness ratio (weekly): 2096 euros per g/dl of Hb for DA, with a CI ranging from − 1427.27 euros per g/dl (less effective and more costly) to 604.62 euros per mg/dl.
- Incremental cost-effectiveness ratio (biweekly): 1560 euros per g/dl of Hb for DA, with a CI ranging from − 1063.64 euros per g/dl (less effective and more costly) to 450 euros per g/dl.

In both scenarios, rHuEPO proved to be more cost-effective. Additionally, we analyzed the cost-effectiveness of switching therapy from rHuEPO to DA, considering the same schedules as above and an observed effectiveness difference of + 0.92 mg/dl of Hb in the DA group (95% CI 0.43–1.41) over 24 weeks:

- Weekly switching: DA demonstrates an incremental cost-effectiveness ratio of 341.74 euros per g/dl of Hb (95% CI 223.4–731.63 euros per g/dl of Hb) with one administration per week for DA and three per week for rHuEPO.
- Biweekly switching: DA demonstrated an incremental cost-effectiveness ratio of 254.35 euros per g/dl of Hb (95% CI 165.96–544.19 euros per g/dl of Hb) with one administration every 2 weeks for DA and one per week for rHuEPO.

Overall, rHuEPO remains the more cost-effective option in both standard and switching scenarios.

## Discussion

The selection of the initial treatment for managing anemia associated with CKD in children remains a topic of debate. The most commonly used therapies, rHuEPO and DA, have not shown definitive superiority in terms of efficacy [9, 16]. A recent meta-analysis conducted in adults with CKD-related anemia similarly concluded that the comparative effects of different ESAs on critical outcomes, such as blood transfusions, mortality, major cardiovascular events, myocardial infarction, stroke, vascular access thrombosis, kidney failure, fatigue, and breathlessness, remain uncertain [29].

Our meta-analysis confirms no significant difference in Hb increase between rHuEPO and DAor in reducing the need for blood transfusion in children with CKD-related anemia after 21–28 weeks of treatment. These findings align with previous studies, such as those by Bruce et al., which highlighted that all types of ESA preparations and administration methods exhibited comparable efficacy [16]. This equivalence in efficacy prompts further discussion about the practical implications of these treatments. The lower frequency of DA administration may benefit patients with poor adherence [30], while the increased injection-site pain associated with DA may pose a challenge [31].

However, some studies performed on the adult population have shown that the use of a prefilled, single-use autoinjector pen can reduce pain and improve patient satisfaction compared to other methods of darbepoetin administration [32, 33].

Additionally, the financial burden of these treatments is noteworthy, especially in resource-limited settings. Our cost-effectiveness analysis indicates that DA incurs higher production costs for comparable effectiveness when compared to rHuEPO, as suggested in other studies [22, 23]. This supports the consideration of rHuEPO as the first-line treatment for children with CKD-related anemia.

However, switching from rHuEPO to DA shows a significant increase in Hb levels of approximately 1 mg/dl (0.93 mg/dl, CI 0.53–1.33 mg/dl), suggesting that DA may be a viable option when rHuEPO fails to achieve adequate Hb control, even at the maximum dose with three weekly administrations. In this context, our cost-effectiveness analysis demonstrates that the use of DA in a switch strategy is associated with an incremental cost-effectiveness ratio of approximately 340 euros per g/dl of Hb over 24 weeks of therapy. Given the significant impact of anemia on morbidity and mortality in children with CKD [4, 34, 35], this cost may be acceptable. However, Cleemput et al. caution that incremental cost-effectiveness ratio alone may not adequately capture the value of an intervention. The threshold value against which incremental cost-effectiveness ratios are evaluated varies across healthcare systems and over time, underscoring a limitation of relying solely on this measure [36]. Another crucial aspect is patient preference regarding treatment administration. The choice between a once-weekly injection and a regimen requiring two to three injections per week may significantly influence both physician recommendations and patient adherence. Given the long-term nature of ESA therapy, treatment convenience and patient comfort should be key considerations in selecting the most appropriate agent.

Our analysis has some limitations. The inclusion of patients at different stages of CKD, ranging from stage 3 to kidney failure requiring dialysis, results in a highly heterogeneous study population. The severity and underlying causes of anemia, as well as the need for ESA therapy, differ considerably between patients with stable stage 3 CKD and those undergoing dialysis for kidney failure. This variability introduces challenges in interpreting the results and drawing generalized conclusions. Moreover, several factors known to complicate anemia management were not accounted for in our analysis. These include chronic inflammation, iron supplementation, suboptimal control of CKD-mineral bone disorder (CKD-MBD), poor adherence to therapy, and the specific characteristics of the underlying primary disease. The omission of these variables may limit the applicability of our findings to real-world clinical practice. Furthermore, the heterogeneity of study designs represents an additional limitation in synthesizing the overall evidence. While all studies shared a focus on Hb profiles and treatment efficacy, differences in criteria for assessing Hb levels after 6 months revealed variability in the methodologies employed. The inclusion of RCTs proved challenging due to the complexities of conducting such trials in children; only two of the selected studies were RCTs. Furthermore, in the analysis of the switch from rHuEPO to DA, the absence of a control group introduces a potential bias and lowers the quality of evidence. Moreover, the variability in the conversion ranges from short-acting EPO to darbepoetin (200–250 IU of EPO for 1 µg of darbepoetin), which may affect the generalizability and precision of the cost-effectiveness analysis. Future research should address these limitations by standardizing Hb assessment criteria and incorporating more rigorous study designs, such as RCTs, to strengthen the evidence base. Additionally, economic evaluations of these therapies should account for healthcare system differences to better inform treatment strategies for managing anemia in pediatric CKD patients. A key limitation of the cost-effectiveness analysis is the variation in reimbursement models, particularly in the US, where the bundled payment model for dialysis can affect both cost and care quality. This model provides a fixed payment for dialysis, including all associated services, regardless of actual costs. Consequently, cost-effectiveness data may be skewed due to differences in healthcare systems, resources, and reimbursement rates, which should be considered when interpreting the results, as they may not apply universally across regions with varying payment structures [36, 37].

## Conclusions

No significant differences were observed in maintaining adequate Hb levels or in the need for blood transfusions among children with CKD-related anemia treated with DA or rHuEPO over a 21–28 week period. Based on its cost-effectiveness, we suggest initiating treatment with rHuEPO. However, switching to DA should be considered after 21–28 weeks if an adequate Hb level is not achieved with rHuEPO therapy.

## Declarations

### Data availability statement

The data underlying this manuscript are available in the article and in its online supplementary material.
